# Particulate Matter Exposure and the Changes in Immune Biomarkers: Effects of Biyeom-Go on the Nasal Mucosa of Patients with Allergic Rhinitis and a Particulate Matter-Treated Mouse Model

**DOI:** 10.1155/2022/4259669

**Published:** 2022-03-26

**Authors:** Bongkyun Park, Byoung-Kab Kang, Ae-Ran Kim, Jung In Kang, Dong‐Hyo Lee, Chang Sop Yang, Young Sook Kim, Chan-Sik Kim

**Affiliations:** ^1^KM Convergence Research Division, Korea Institute of Oriental Medicine, Daejeon 34054, Republic of Korea; ^2^KM Science Research Division, Korea Institute of Oriental Medicine, Daejeon 34054, Republic of Korea; ^3^Clinical Research Coordinating Team, Korea Institute of Oriental Medicine, Daejeon 34054, Republic of Korea; ^4^Department of Ophthalmology, Otolaryngology, and Dermatology, College of Korean Medicine, Woo‐Suk University, 46 Eoeun‐ro, Wansangu, Jeonju‐si, Jeollabuk‐do, Wanju gun 54987, Republic of Korea

## Abstract

This study was to investigate the effects of Biyeom-go (BYG, an herbal formula) on immune biomarkers present in the nasal mucosa of patients with allergic rhinitis under exposure to particulate matter 2.5 (PM2.5), and on changes in goblet cells and immune biomarkers in mice under exposure to Korea diesel particulate matter (KDP20). Thirty patients showing characteristic allergic rhinitis symptoms were enrolled in Jeonju-si, Korea, and treated with BYG thrice a day for four weeks. Changes in the expression of immune biomarkers (interleukin 4 (IL-4), IL-5, IL-8, IL-13, IL-33, and thymic stromal lymphopoietin (TSLP) mRNA), total nasal symptom scores (TNSS), mini-rhinitis-specific quality of life questionnaire (RQLQ) results, and visual analog scale scores were evaluated after 4 weeks of treatment. Additionally, the difference in PM2.5 concentrations in the air in Jeonju-si, Korea (November, 2019 ∼ March, 2020), was analyzed to determine the change in TNSS. KDP20 (100 *μ*g/mL) was exposed to C57BL/6 mice for 10 days; 0.05% Nasonex (a positive control, mometasone furoate), or BYG was administrated for 5 days twice a day. The expression of inflammatory factors was detected via qRT-PCR using nasopharynx tissue samples of mice. BYG treatment was found to be associated with significant improvement in total nasal symptoms, especially itching and sneezing (*p* < 0.0001), and mini-RQLQ after 4 weeks. IL-8 (*p* < 0.01), IL-33 (*p* < 0.01), and TSLP (*p* < 0.001) expression levels decreased after BYG treatment. In mice, administration of BYG reduced the number of goblet cells increased through KDP20 treatment. KDP20-induced immune biomarkers (IL-33, TSLP, tumor necrosis factor alpha, and IL-8) were also significantly downregulated in the nasopharynx tissue after BYG treatment. Therefore, BYG may show therapeutic effects against allergic rhinitis in humans, and it was confirmed that the expression of PM-induced inflammatory factors in mice was decreased *via* BYG treatment.

## 1. Introduction

Particulate matter (PM) has been identified as an important environmental risk factor for allergies, respiratory diseases, and cardiovascular morbidity and mortality [1]. PM is categorized based on the aerodynamic diameter of the particles. PM with a diameter of 10 *μ*m or less among the total suspended particles in the air is defined as PM10, and PM with a diameter of <2.5 *μ*m is classified as PM2.5. Fine PM (PM2.5) penetrates further into the human body than other PM, causing various inflammatory reactions in the human body. It is associated with increased mortality, cardiovascular effects, respiratory effects, and cancer incidence [1]. The association of PM with health disorders has been reported in several PM health hazard assessment studies. PM2.5 and PM10 are associated with a significant increase in-hospital admissions for respiratory diseases based on increasing PM levels in Korea [2].

Exposure to PM modulates the airway epithelium and promotes the production of several cytokines, including interleukin 1 (IL-1), IL-6, IL-8, IL-25, IL-33, tumor necrosis factor-alpha (TNF-*α*), thymic stromal lymphopoietin (TSLP), and granulocyte-macrophage colony-stimulating factor. PM-induced type 2-promoting cytokines are important mediators in the acute and aggravating effects of PM on airway inflammation [3]. These cytokines are targeted in the treatment of allergic airway inflammation and asthma. High concentrations of PM2.5 upregulate TNF-*α* and the helper T cell 2 (Th2)-mediated cytokines IL-4 and IL-10. Exposure to PM2.5 leads to the loss of barrier function in humans, and it is associated with allergic rhinitis development via penetration of the mucus barrier over the mucociliary epithelium. Moreover, PM2.5 directly affects the cardiovascular system and pulmonary tissues [4]. Oxidative stress, inflammatory response, and genotoxicity have been proposed to be the major causal factors involved in the mediation of PM-induced effects on pulmonary health outcomes [5]. Intranasal herbal treatment using ointments, drops, and sprays is widely performed for treating rhinitis in East Asian and European countries [6–8].

Biyeom-go (BYG), a herbal ointment, is composed of the Korean Medicine herbal prescription, Hwanglyeonhaedok-tang with *Sophorae Radix* and *Glycyrrhizae Radix* et *Rhizoma* added. It is clinically used in Korea to improve nasal symptoms associated with rhinitis [9]. Prospective observational studies have shown that Biyeom-go (BYG) helps manage rhinitis symptoms and improves rhinitis-associated quality of life [6, 9]. IL-2 levels are found to increase, and IL-8, chemokine (C-C motif) ligand (CCL) 5, chemokine (C-X-C motif) ligand (CXCL) 9, CCL2, and CXCL10 levels decrease in the nasal lavage fluid after treatment with BYG. To identify the immune biomarkers in rhinitis that are associated with the effects of PM and BYG, we examined the effects of BYG on the changes in immune biomarkers in patients with rhinitis and PM-exposed mice in this study.

## 2. Materials and Methods

### 2.1. Participants and Ethical Considerations

Patients who were provided with and used BYG and those that met the following criteria were included: (1) age over 19 years, (2) more than 2 symptoms of allergic rhinitis (nasal congestion, rhinorrhea, itching, and sneezing), and (3) positive result in the testing for allergen-specific IgEs with the multiple allergen simultaneous test within 12 months. The following conditions were exclusion criteria: pregnancy, breastfeeding, or planning for pregnancy for the next 2 months. This prospective observational clinical study was performed at the Woosuk Korean Medicine Medical Center Jeonju-si, Korea, between October 2019 and March 2020. BYG was manufactured by the Dispensary Pharmacy of Woosuk Korean Medicine Medical Center, and the quality was controlled [9]. Patients were provided with BYG that was used *via* a cotton swab thrice a day for 4 weeks. The protocol was approved by the Institutional Review Board of the Woosuk Korean Medicine Medical Center (WSOH IRB H1810-04-06).

### 2.2. Concentrations of PM2.5 in the Air of Jeonju-Si

Surface PM2.5 concentrations in Jeonju-si, Korea, were obtained every hour from the AIR Korea data archive (https://www.airkorea.or.kr) and from the National Ambient Air Quality Monitoring Information System. The concentration of PM2.5 (*μ*g/m^3^) was measured at five monitoring sites in Jeonju-si, between November 2019 and March 2020. Locations of the monitors are shown in the supplementary material (Table S1 and Figure S1).

### 2.3. Collection of Nasal Swabs

To examine the changes in the mRNA expression of immune factors, 60 nasal fluid samples from 30 patients were collected at the initial and fifth visits. The nasal swabs were collected using sterile cotton swabs (25-3306-U TT FDNA, Guilford, ME, USA), which were used to sample the internal anterior walls of both nostrils and were then placed in the provided transport tube that contained a viral transport media-soaked foam pad at the base. Within 24 h of collection, the nasal swabs were stored at −80°C until analysis.

### 2.4. Animal Experiments

Six-week-old male C57BL/6 mice were purchased from Orient Bio (Seoul, Korea). Mice in the normal control group (NOR) were subjected to intranasal administration of phosphate-buffered saline for 10 days, and mice exposed to Korea diesel particulate matter (KDP20) for 10 days were randomly allocated to three groups (*n* = 10, each group): (1) KDP20-treated mice were administered with a vehicle (KDP20), (2) KDP-treated mice were administered with 0.05% mometasone furoate (positive control, Nasonex), and (3) KDP20-treated mice were administered with 100% BYG (BYG). BYG and Nasonex were nasally administered twice a day. The animal experiments were approved by the Institutional Animal Care and Use Committee (IACUC approval no. 20–027).

### 2.5. Immunohistochemistry

All samples were fixed in 10% formalin for 24 h and then embedded in paraffin. Sections of 5 *μ*m thickness were cut and stained using periodic acid-Schiff (PAS; ab150680; Abcam, Cambridge, United Kingdom) and Alcian blue (ab15662; Abcam). Images of the tissues were captured and observed blindly via light microscopy (BX43; Olympus Lifescience, Tokyo, Japan).

### 2.6. Quantitative Real-Time Polymerase Chain Reaction (qRT-PCR)

An RNA extraction kit (Invitrogen, Carlsbad, CA, USA) was used to isolate RNA from human nasal secretions from the back of the nose and from the nasopharynx tissues of mice. cDNA was synthesized from the isolated RNA (1 mg/mL) using a SuperScript II kit (Bio-Rad, Hercules, CA, USA). The cDNA was amplified via q-PCR using specific primers listed in Table S2 using a thermocycler (Bio-Rad).

### 2.7. Statistical Analysis

All analyses were performed by an independent statistician using SAS version 9.4 (SAS Institute, Inc., Cary, NC, USA) and GraphPad Prism 7.05 (GraphPad Software, San Diego, CA, USA). Data are presented as mean ± standard deviation (SD). Differences between groups were analyzed using the unpaired *t*-test or Wilcoxon rank-sum test. One-way ANOVA was used to compare multiple groups. Values of *p* < 0.05 were considered statistically significant.

## 3. Results

### 3.1. Changes in Air Pollution in Jeonju-Si

During the study period, Jeonju-si experienced a daily average ambient PM2.5 concentrations of 30.88 ± 11.69 *μ*g/m^3^, which was lower than the concentration recommended by the World Health Organization (35 *μ*g/m^3^). The air was relatively clean due to the reduced social activity after the Coronavirus disease 2019 (COVID-19) outbreak. To determine the effects of BYG on PM-induced rhinitis, we performed statistical analyses on the ecological association between immune biomarkers and PM2.5 concentrations. Patient characteristics are summarized in Table S3.

### 3.2. Effects of BYG Treatment on Nasal Symptoms Defined by TNSS and Mini-RQLQ

After BYG treatment, the TNSS and mini-RQLQ exhibited significant improvements from baseline at each visit (Table 1). Significant differences in TNSS and mini-RQLQ were found between baseline and week 4 in nasal congestion, rhinorrhea, itching, and sneezing. The mean TNSS score was reduced from 6.1 ± 2.3 to 3.4 ± 2.2 (*p* < 0.0001), and the mini-RQLQ score was reduced from 38.6 ± 11.9 to 19.3 ± 12.8.

The nasal endoscopy index significantly improved after 4 weeks of treatment compared to that at baseline (Table 2). As the BYG treatment progressed, nasal discharge and atrophy edema did not improve compared to those at baseline. Particularly, the color and dryness dampness were significantly improved after BYG treatment. We then compared the relevance between air quality (PM2.5 < 35 *μ*g/m^3^, PM2.5 > 35 *μ*g/m^3^) and the change in TNSS after BYG treatment. The mean TNSS in the PM2.5 > 35 *μ*g/m^3^ group was slightly higher than that in the PM2.5 < 35 *μ*g/m^3^ group; after treatment with BYG, TNSS was significantly reduced regardless of the concentration of PM2.5 (Table 3). However, there was no difference in the improvement of nasal symptoms based on the concentration of PM2.5 (Table 4).

### 3.3. Effects of BYG Treatment on the Expression of Immune Biomarkers

**Figure 1:**
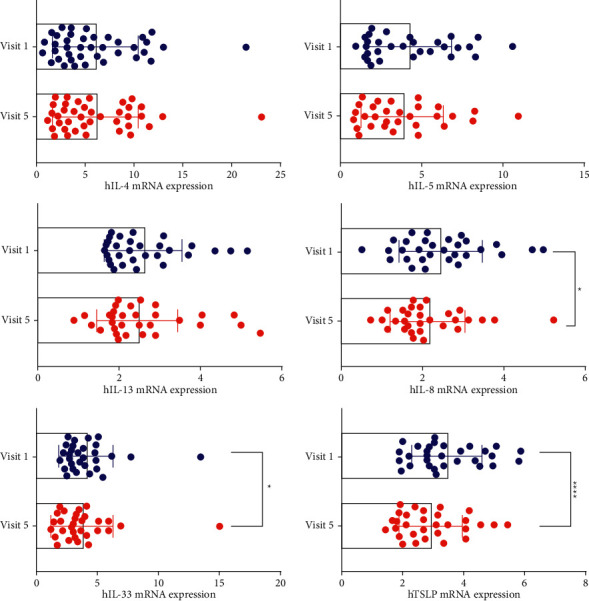
Expression of human IL-4, IL-5, IL-8, IL-13, IL-33, and TSLP mRNA in the human nasal mucosa. mRNA gene expression was measured via real time-polymerase chain reaction and presented as relative fold-changes to visit 1 sample. Data are presented as the mean ± standard deviation (*n* = 30). ^*∗*^*p* < 0.01^*∗∗∗*^*p* < 0.001. IL-4, interleukin 4; TSLP, thymic stromal lymphopoietin.

We compared the mRNA expression levels of immune biomarkers in the nasal mucosa of 30 patients before and after treatment with BYG. The levels of IL-8 (*p* < 0.01), IL-33 (*p* < 0.01), and TSLP mRNA (*p* < 0.001) were significantly reduced after BYG treatment. However, there were no changes in the mRNA expression of IL-4, IL-5, and IL-13 (Figure 1).

### 3.4. Effects of BYG Treatment on Expression of Goblet Cells and Inflammatory Factors in PM-Treated Mice

**Figure 2:**
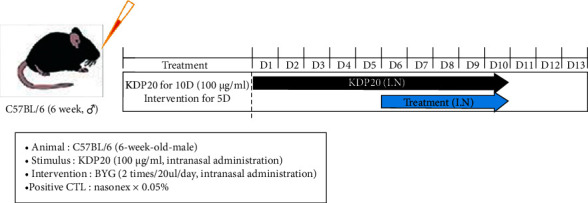
Generation of a PM-induced animal model and drug administration. KDP20, Korea diesel particulate matter; BYG, Biyeom-go; Nasonex, positive control; I.N., intervention; CTL, control.

In the aforementioned human experiment, the effect of BYG was confirmed; however, we could not evaluate the changes attributed to PM2.5 because the air quality had improved owing to reduced social activity during the COVID-19 outbreak. Therefore, we examined the effects of BYG in a KDP20-induced nasal inflammation mouse model. BYG and Nasonex were intranasally administered twice a day for 5 days (Figure 2).

**Figure 3:**
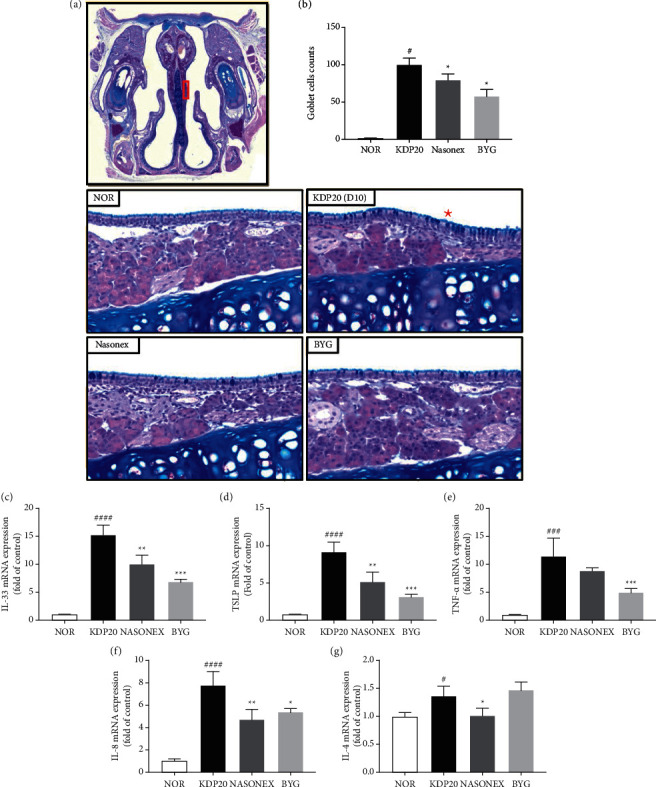
Effects of BYG and Nasonex treatment on expression of goblet cells and inflammatory factors in KDP20-induced mice. (a) Generation of a KDP-induced animal model and drug administration. (b) Representative images of goblet cells with PAS staining of nasal tissues in KDP20-treated mice. PAS-positive cells were distributed in the nasal epithelium. (c–g) mRNA expression of immune biomarkers. NOR, negative control; KDP20, Korea diesel particulate matter; BYG, Biyeom-go; Nasonex, positive control; PAS, periodic acid-Schiff; IL-33, interleukin 33; TSLP, thymic stromal lymphopoietin; TNF-*α*, tumor necrosis factor-alpha.

The number of mucus-secreting goblet cells significantly increased in the epithelium on one side of the nasal septum (red asterisk) after exposure to KDP20 (Figure 3(a)). However, treatment with BYG and Nasonex (positive control) remarkably reduced the number of goblet cells compared to that in the KDP20-treated group. Exposure to PM increases the levels of inflammatory cytokines associated with respiratory disease [10]. In particular, alarmin cytokines such as IL-25, IL-33, and TSLP are strongly associated with the development of rhinitis due to PM exposure [11, 12]. Therefore, we evaluated the effects of BYG on the expression of inflammatory cytokines. qPCR analysis revealed that the mRNA expression of IL-33, TSLP, TNF*-α*, IL-8, and IL-4 was significantly increased *via* KDP20 treatment; however, treatment with BYG markedly inhibited the upregulation of the inflammatory cytokines except for IL-4 (Figures 3(c)–3(g)).

## 4. Discussion

Allergic rhinitis is an inflammatory disorder to the nasal mucosa caused by allergen exposure that causes IgE-mediated inflammation. Clinically, it is characterized by four main symptoms such as rhinorrhea, sneezing, nasal itching, and nasal congestion and is associated with pathogenesis with asthma and nasal polyps [13]. The mucosal immune system in humans is poorly studied; however, it has proven to be important in establishing an effective immune response to airborne pathogens and toxic pollutants such as PM, by stimulating Th1/Th2 cells, cytotoxic T lymphocyte production, plasma, and B cells [14]. It has been found that BYG treatment is beneficial in the management of rhinitis symptoms and rhinitis-associated quality of life by modulating the immune response [9]. Here, we examined the effects of BYG on immune biomarkers in patients with rhinitis and mice exposed to PM.

PM induces the activation of pathogenic immune cells in mucosal tissues with disrupted barriers, which then migrate to the affected organs such as brain, lung, and gut [15]. It is associated with increased disease activity in multiple sclerosis together with an increase in CCR6^+^CD4^+^ T cells with migratory properties [16]. Epidemiological studies suggest that exposure to PM10 increases the risk of developing allergic rhinitis [17]. A previous study shows that PM2.5 causes defects in the nasal epithelial barrier in noninflamed nasal biopsy samples of patients with sinusitis [18]. A significant association between PM2.5 exposures at birth and the development of allergic rhinitis at the age of 7–8 has been shown in a meta-analysis of 6 birth cohorts in Canada [19]. Exposure to PM2.5 exacerbates allergic rhinitis in mice by increasing DNA methylation of the interferon-gamma gene promoter in CD4+ T cells *via* the extracellular signal-regulated kinase-DNA methyltransferase pathway [20].

The PM2.5 concentrations were >35 *μ*g/m^3^ in the air in Jeonju-si, Korea, for only a few days during the study period; therefore, it was difficult to directly determine the association of PM2.5 concentrations with nasal symptoms in allergic rhinitis. Also, the mean TNSS value in the PM2.5 > 35 *μ*g/m^3^ group (*N* = 15) was not significantly different from that in the PM2.5 < 35 *μ*g/m^3^ group (*N* = 15). However, BYG treatment (*N* = 30) for 4 weeks was associated with a significant improvement in total nasal symptoms, especially itching and sneezing, and an improvement in mini-RQLQ was determined based on downregulation of IL-8 and alarmin cytokines such as IL-33 and TSLP in the nasal mucosa. IL-33 functions as an alarm signal that is rapidly released from cells upon cellular damage or stress [21]. TSLP belongs to the IL-2 cytokine family and promotes Th2 responses in the lung- and skin-specific allergic disorders [22]. TSLP expression in the nasal epithelial cells of patients with allergic rhinitis is found to be significantly greater than that in the nasal mucosa of patients with nonallergic rhinitis [23]. Previous studies show that PM affects the distribution of occludin and the alveolar epithelial cells in *ex vivo* humans and rats.

Although it was not possible to directly compare the effects on PM10 and PM2.5, our results showed that KDP20 exposure for 10 days significantly increased the expression of immune biomarkers IL-33, TSLP, TNF-*α*, and IL-8 in the nasopharynx tissue, suggesting that PM represents a causative agent of rhinitis in animals. Moreover, PM induced the secretion of mucin in goblet cells that increased in number. The findings of this study suggest that BYG may improve nasal symptoms and may be used to develop treatments for PM-induced inflammation in rhinitis.

## Figures and Tables

**Table 1:** The change of TNSS and mini-RQLQ for 4 weeks.

Nasal symptoms	Baseline	Week 1	Week 2	Week 3	Week 4	Difference (baseline-4 weeks)	*p* value
Nasal congestion	1.7 ± 0.7	1.4 ± 0.7^*∗*^	1.5 ± 0.8	1.3 ± 0.8^*∗*^	1.1 ± 0.8^*∗*^	0.6 ± 0.9	**0.0011**
Rhinorrhea	1.6 ± 0.7	1.6 ± 0.8	1.4 ± 0.7	1.3 ± 0.7^*∗*^	1.1 ± 0.8^*∗*^	0.5 ± 0.9	**0.0067**
Itching	1.3 ± 0.9	1.0 ± 0.8	0.8 ± 0.7^*∗*^	0.7 ± 0.7^*∗∗*^	0.5 ± 0.6^*∗∗*^	0.7 ± 0.8	**<0.0001**
Sneezing	1.5 ± 0.8	1.1 ± 0.8^*∗*^	0.9 ± 0.7^*∗∗*^	0.6 ± 0.6^*∗∗*^	0.6 ± 0.7^*∗∗*^	0.9 ± 0.8	**<0.0001**
TNSS	6.1 ± 2.3^a^	5.0 ± 2.2^*∗*^^b^	4.6 ± 2.1^*∗*^^bc^	3.8 ± 2.0^*∗∗*^^cd^	3.4 ± 2.2^*∗∗*^^d^	2.7 ± 2.6	**<0.0001**
Mini-RQLQ	38.6 ± 11.9^a^	27.4 ± 10.1^*∗∗*^^b^	25.6 ± 10.2^*∗∗*^^b^	24.0 ± 11.6^*∗∗*^^bc^	19.3 ± 12.8^*∗∗*^^c^	19.3 ± 12.8	**<0.0001**

TNSS, total nasal symptom score; mini-RQLQ, mini‐rhinoconjunctivitis quality of life questionnaire. The values are expressed as the mean ± SD. RM ANOVA ^*∗*^*p* < 0.05; ^*∗∗*^*p* < 0.001.

**Table 2:** The change of TNSS and mini-RQLQ.

Nasal symptoms	Baseline	4 weeks	Difference (baseline-4 weeks)	*p* value
Nasal congestion	1.7 ± 0.7	1.1 ± 0.8	0.6 ± 0.9	0.0011
Rhinorrhea	1.6 ± 0.7	1.1 ± 0.8	0.5 ± 0.9	0.0067
Itching	1.3 ± 0.9	0.5 ± 0.6	0.7 ± 0.8	<.0001
Sneezing	1.5 ± 0.8	0.6 ± 0.7	0.9 ± 0.8	<.0001
TNSS	6.1 ± 2.3	3.4 ± 2.2	2.7 ± 2.6	<.0001
Mini-RQLQ	38.6 ± 11.9	19.3 ± 12.0	19.3 ± 12.8	<.0001

TNSS, total nasal symptom score; mini-RQLQ, mini‐rhinoconjunctivitis quality of life questionnaire. The values are expressed as the mean ± SD.

**Table 3:** The changes in the nasal endoscopy index.

Nasal endoscopy index	Baseline	4 weeks	Difference (baseline-4 weeks)	*p* value
Left				
Color	0.9 ± 0.4	0.6 ± 0.5	0.3 ± 0.5	**0.0046**
Dryness dampness	0.9 ± 0.3	0.4 ± 0.5	0.5 ± 0.6	**0.0003**
Nasal discharge	0.2 ± 0.4	0.1 ± 0.3	0.1 ± 0.6	0.3746
Atrophy edema	0.1 ± 0.3	0.1 ± 0.3	0 ± 0.4	0.6624
Right				
Color	0.9 ± 0.4	0.5 ± 0.5	0.4 ± 0.5	**0.0001**
Dryness dampness	0.9 ± 0.4	0.4 ± 0.5	0.6 ± 0.6	**<0.0001**
Nasal discharge	0.2 ± 0.4	0.2 ± 0.4	0 ± 0.6	1.0000
Atrophy edema	0.3 ± 0.6	0.2 ± 0.4	0.1 ± 0.5	0.4888
Total	4.5 ± 1.9	2.6 ± 2.0	1.9 ± 2.1	**<0.0001**

Bolded *p* values represent statistically significant values: Baseline vs. 4 weeks.

**Table 4:** Comparison of the difference of PM2.5 air quality and the change of TNSS.

Difference of PM2.5 air quality (baseline-4 weeks)			
Nasal symptom	PM2.5 < 35 *μ*g/m^3^	PM2.5 > 35 *μ*g/m^3^	*p* value^1^
Nasal congestion	0.4 ± 0.74	0.73 ± 0.96	0.2757
Rhinorrhea	0.53 ± 0.83	0.47 ± 1.06	0.8626
Itching	0.87 ± 0.74	0.60 ± 0.91	0.4955
Sneezing	0.87 ± 0.74	0.93 ± 0.96	0.8412
TNSS	2.73 ± 1.94	2.73 ± 3.22	0.9008

TNSS, total nasal symptom score. The values are expressed as the mean ± SD. *p* value^1^ was calculated through the nonparametric statistics (Wilcoxon rank-sum test, *n* = 15 each).

## Data Availability

The datasets analyzed during the current study are available from the corresponding author upon reasonable request.

## Supplementary Materials

Supplementary Materials Table S1: list of the air quality index for 4 weeks. Table S2: sequences of primers used for qRT-PCR. Table S3: demographic and blood data for the participants. Figure S1: days of PM2.5 > 35 *μ*g/m^3^ from October 2019 to March 2020 in Jeonju-si, South Korea.
