# Supplementary material for: Particulate Matter Exposure and the Changes in Immune Biomarkers: Effects of Biyeom-Go on the Nasal Mucosa of Patients with Allergic Rhinitis and a Particulate Matter-Treated Mouse Model
Source: Evid Based Complement Alternat Med. 2022 Mar 26;2022:4259669. doi: 10.1155/2022/4259669 (PMC8976652; doi:10.1155/2022/4259669)
Supplement: Supplementary Materials — Table S1: list of the air quality index for 4 weeks. Table S2: sequences of primers used for qRT-PCR. Table S3: demographic and blood data for the participants. Figure S1: days of PM2.5 > 35 μg/m3 from October 2019 to March 2020 in Jeonju-si, South Korea. [file 4259669.f1.docx]

**Table.S1. List of air quality index for 4 weeks**

| **Date** | **Oct.2019** | **Nov. 2019** | **Dec. 2019** | **Jan. 2020** | **Feb. 2020** | **Mar. 2020** |
| --- | --- | --- | --- | --- | --- | --- |
| 1 | 36.4 | 48.3 | 39.5 | 48.3 | 55 | 40 |
| 2 | 14.4 | 47.8 | 23.5 | 47.8 | 63.5 | 35 |
| 3 | 29.2 | 49 | 16.5 | 49 | 30 | 36 |
| 4 | 50 | 38.3 | 25 | 38.3 | 26.7 | 26 |
| 5 | 51.6 | 43 | 20.5 | 43 | 17.3 | 33 |
| 6 | 26.8 | 31.8 | 21.3 | 31.8 | 19.7 | 30 |
| 7 | 21.4 | 31.3 | 29.5 | 31.3 | 31 | 31 |
| 8 | 36.2 | 29 | 35.8 | 29 | 36 | 36 |
| 9 | 28.6 | 36.3 | 37 | 36.3 | 26 | 62 |
| 10 | 36.3 | 31 | 48 | 31 | 45 | 43 |
| 11 | 52.5 | 21 | 40.5 | 21 | 45 | 19 |
| 12 | 37.8 | 26.8 | 20.5 | 26.8 | 23.5 | 19 |
| 13 | 33.4 | 27.3 | 26.3 | 27.3 | 36.5 | 9 |
| 14 | 42.2 | 14.5 | 33.3 | 14.5 | 51 | 16 |
| 15 | 44.2 | 16.5 | 32 | 16.5 | 42 | 18 |
| 16 | 43.2 | 24 | 23.8 | 24 | 15 | 13 |
| 17 | 67.6 | 23 | 21 | 23 | 16 | 19 |
| 18 | 33.8 | 21.3 | 34.8 | 21.3 | 21 | 26 |
| 19 | 37.6 | 17.8 | 22 | 17.8 | 22 | 19 |
| 20 | 46.8 | 19 | 29 | 19 | 38 | 11.5 |
| 21 | 39.4 | 27 | 38 | 27 | 42 | 20 |
| 22 | 30.6 | 24.8 | 34.7 | 24.8 | 38 | 28.5 |
| 23 | 32 | 26.3 | 39.3 | 26.3 | 18 | 27.5 |
| 24 | 31.6 | 26.5 | 52 | 26.5 | 23 | 42 |
| 25 | 37.8 | 22.5 | 45.3 | 22.5 | 15 | 34 |
| 26 | 34.2 | 27.3 | 33 | 27.3 | 17 | 12.3 |
| 27 | 31 | 30.5 | 21 | 30.5 | 41 | 11.8 |
| 28 | 36.3 | 42.8 | 26 | 42.8 | 28 | 10.8 |
| 29 | 74.4 | 32.3 | 26.7 | 32.3 | 20 | 18.3 |
| 30 | 41 | 31.5 | 18.7 | 31.5 |  | 20.3 |
| 31 | 60.8 |  | 18.5 | 35.5 |  | 18 |
| **Days (PM2.5 >35 μg/m^3^)** | **19** | **7** | **8** | **7** | **11** | **4** |

| **Table S2**. Sequences of primers used for qRT-PCR   \| Gene \| Sequence \| \|  \| 5’ → 3’ \| \| --- \| --- \| --- \| --- \| --- \| \| **mIL-33** \| Forward \| ATG GGA AGA AGC TGA TGG TG \| \| \| \| Reverse \| CCG AGG ACT TTT TGT GAA GG \| \| \| \| **mTSLP** \| Forward \| AGG CTA CCC TGA AAC TGA G \| \| \| \| Reverse \| GGA GAT TGC ATG AAG GAA TAC C \| \| \| \| **mTNF-α** \| Forward \| AAG CCT GTA GCC CAC GTC GTA \| \| \| \| Reverse \| GGC ACC ACT AGT TGG TTG TCT TTG \| \| \| \| **mIL-8** \| Forward \| CTA GGC ATC TTC GTC CGT CC \| \| \| \| Reverse \| TTC ACC CAT GGA GCA TCA GG \| \| \| \| **mIL-4** \| Forward \| ACA GGA GAA GGG ACG CCA T \| \| \| \| Reverse \| GAA GCC GTA CAG ACG AGC TCA \| \| \| \| **mGAPDH** \| Forward \| GCA CAG TCA AGG CCG AGA AT \| \| \| \| Reverse \| GCC TTC TCC ATG GTG GTG AA \| \| \| \| **hIL-4** \| Forward \| AAC AGC CTC ACA GAG CAG AAG AC \| \| \| \| Reverse \| GTG TTC TTG GAG GCA GCA AAG \| \| \| \| **hIL-5** \| Forward \| TGG AGC TGC CTA CGT GTA TGC \| \| \| \| Reverse \| GCA GTG CCA AGG TCT CTT TCA C \| \| \| \| **hIL-8** \| Forward \| CTG GCC GTG GCT CTC TTG \| \| \| \| Reverse \| TTA GCA CTC CTT GGC AAA ACT G \| \| \| \| **hIL-13** \| Forward \| GCT CCA GCA TTG AAG CAG TG \| \| \| \| Reverse \| CGT GGC AGA CAG GAG TGT T \| \| \| \| **hIL-33** \| Forward \| AAT CAG GTG ACG GTG TTG \| \| \| \| Reverse \| ACA CTC CAG GAT CAG TCT TG \| \| \| \| **hTSLP** \| Forward \| TAG CAA TCG GCC ACA TTG CCT \| \| \| \| Reverse \| GAA GCG ACG CCA CAA TCC TTG \| \| \| \| **hGAPDH** \| Forward \| ACA TCA TCC CTG CAT CCA CT \| \| \| \| Reverse \| GGG AGT TGC TGT TGA AGT CA \| \| \|   mIL, mouse interleukin; mTSLP, thymic stromal lymphopoietin; mTNF-a, mouse tumor necrosis factor; mGAPDH, glyceraldehyde 3 phosphate dehydrogenase.    **Table S3.** Demographic and blood data for the participants | | |
| --- | --- | --- | --- | --- | --- | --- | --- | --- | --- | --- | --- | --- | --- | --- | --- | --- | --- | --- | --- | --- | --- | --- | --- | --- | --- | --- | --- | --- | --- | --- | --- | --- | --- | --- | --- | --- | --- | --- | --- | --- | --- | --- | --- | --- | --- | --- | --- | --- | --- | --- | --- | --- | --- | --- | --- | --- | --- | --- | --- | --- | --- | --- | --- | --- | --- | --- | --- | --- | --- | --- | --- | --- | --- | --- | --- | --- | --- | --- | --- | --- | --- | --- | --- | --- | --- | --- | --- | --- | --- | --- | --- | --- | --- | --- | --- | --- | --- | --- | --- | --- | --- | --- | --- | --- | --- | --- | --- | --- | --- | --- | --- | --- | --- | --- | --- | --- | --- | --- | --- | --- | --- | --- | --- | --- |
| **Classification** | **Baseline (visit 1)**  **(n=30)** | **4 weeks (visit 5)**  **(n=30)** |
| Sex (males/female) | 15/15 | 15/15 |
| Age | 35.2±10.1 | - |
| Height (cm) | 167.2±8.6 | - |
| Weight (kg) | 66.4±15.2 | - |
| BMI (kg/m2) | 23.6±4.3 | - |
| Blood Pressure (mmHg)  Systolic  Diastolic | 121.1±13.2  70.5±10.8 | 122.2±10.3  72.3±10.2 |
| Pulse (beats/min) | 78.9±13.1 | 82.2±13.4 |
| Body Temp. (℃) | 36.6±0.2 | 36.5±0.2 |
| WBC (K/uL)  Eosinophil (%)  Neutrophil (%)  Basophil (%)  Monocyte (%)  Lymphocyte (%) | 6.5±1.4  2.5±1.7  55.2±8.5  0.8±0.5  7.1±1.7  34.3±7.8 | 6.1±1.1  2.6±1.7  54.3±8.5  0.8±0.5  7.6±1.9  34.6±7 |
| RBC (K/uL) | 4.9±0.4 | 4.8±0.4 |
| Hemoglobin, Hb (g/dl) | 14.2±1.5 | 14.04±1.28 |
| Hematocrit, Hct (%) | 43.5±4 | 43.2±3.7 |
| ESR (mm/hr) | 3.6±4.3 | 3.4±4.3 |
| Platelet (K/uL) | 241.1±65 | 235±59 |
| Total IgE (IU/mL) | 285±477.1 | 277.5±471.5 |
| Eosinophil count (/uL) | 164±128 | 160±136 |

BMI, body mass index; WBC, white blood cell; RBC, red blood cell; ESR erythrocyte sedimentation rate. The values are expressed as the mean±SD.

**Figure S1.**


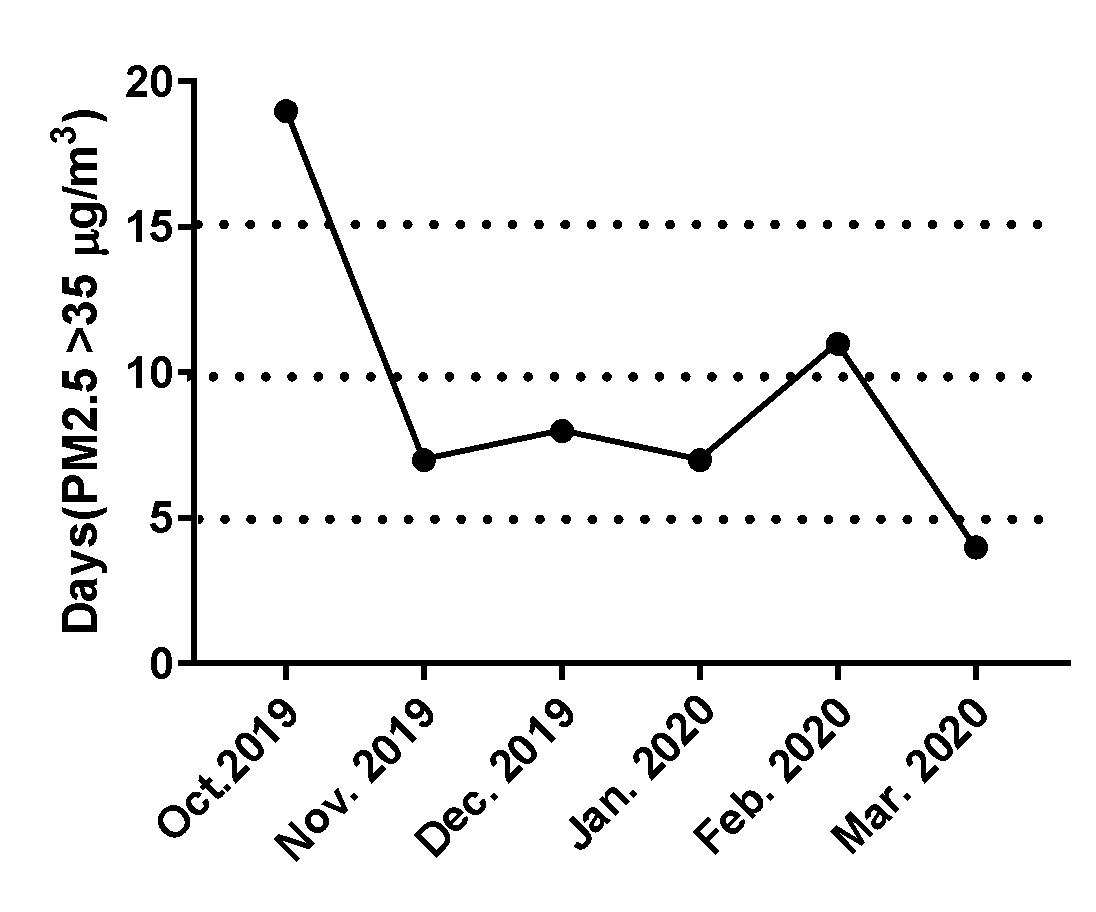


The days of PM2.5 >35 μg/m^3^ from October 2019 to March 2020 in Jeonju-ci, South Korea

**Table 1.** The change of TNSS and mini-RQLQ for 4 weeks

**Table 2.** The change of TNSS and mini-RQLQ

**Table 3**. The change in nasal endoscopy index

| **Table 4.** Comparison of the difference of PM2.5 air quality and the change of TNSS |
| --- |
